# Supplementary material for: MetaRibo-Seq measures translation in microbiomes
Source: Nat Commun. 2020 Jun 29;11:3268. doi: 10.1038/s41467-020-17081-z (PMC7324362; doi:10.1038/s41467-020-17081-z)
Supplement: Supplementary file 10 — Supplementary Data 7 [file 41467_2020_17081_MOESM10_ESM.zip › File2/Confidence_High_Taxonomy/51874_out.krona.html]

Javascript must be enabled to view this page.

members
magnitude
magnitudeUnassigned
count
unassigned
taxon
rank

51874\_out

1

superkingdom
2759
1

kingdom
1
4751

subkingdom
1
451864

1
4890
phylum

147538
1
subphylum

1
147550
class

222544
1
subclass

5139
1
order

35718
1
family

genus
1
2609811

2587410
1
species

SRS143450\_contig\_number\_21817
